# Supplementary material for: Addressing data management and analysis challenges in viral genomics: The Swiss HIV cohort study viral next generation sequencing database
Source: PLOS Digit Health. 2025 Apr 21;4(4):e0000825. doi: 10.1371/journal.pdig.0000825 (PMC12011223; doi:10.1371/journal.pdig.0000825)
Supplement: Attachment — Submitted filename: point_by_point_reply_revision.docx [file pdig.0000825.s002.docx]

**Review Comments in green**

**Author replies in yellow**

# Reviewer #1:

The manuscript by Zeeb et al. (the reviewer misspelled the first authors last name as Zeev) offers an in-depth exploration of how HIV NGS data are processed and managed within the Swiss HIV Cohort Study. The authors have previously contributed several notable studies using NGS data, as highlighted in Table 2. While certain sections provide an excess of detail unlikely to be of broad interest, other critical topics receive insufficient attention. Furthermore, the specific aims of the manuscript are not clearly articulated.

We thank the reviewer very much for the positive feedback and agree that some sections would benefit from a revision, which we outline in detail in the specific comments below. Briefly summarized, we emphasized that the main aim of the manuscript is the design of the NGS database. While we use the database mostly for NGS data from illumina, the database is flexible to manage NGS data from other technologies. In addition, we improved the language at various locations to increase readability. Finally, we added two figures and one table to explain the workflow better.

## 1. Data analysis and amplification methodology:

The approach to data analysis and management depends significantly on the method by which the virus is amplified. Historically, sequencing of the HIV pol gene has relied on sets of well-conserved primers. However, achieving full-genome sequencing, especially of highly variable regions, presents additional challenges. The manuscript does not discuss how the analysis pipeline accounts for the amplification method used to generate whole-genome data. This omission raises doubts about the assertion in the abstract that "NGS can easily achieve near-whole-genome sequence coverage." In fact, several studies reporting full-genome sequencing have employed different strategies, underscoring the complexity involved.

We agree that different amplification methods may result in biases of the obtained whole genome, particularly for highly variable regions such as Env. However, we propose this database as a platform to process NGS data. For most database management processes, the actual data content is not important, the processes can be performed uniformly, independent of the originating amplification method and NGS technology. We achieve this in our database with a flexible approach where each sample has a schema less set of metadata.

General technical challenges in NGS of viral populations we addressed in other works, for example:
Di Giallonardo, Nucleic Acids Res, 2014, https://pubmed.ncbi.nlm.nih.gov/24972832/;

Di Giallonardo, PLoS One, 2013, https://pubmed.ncbi.nlm.nih.gov/24058534/;

Bachmann, Nature communications, 2019 https://pubmed.ncbi.nlm.nih.gov/31324762/;

Chaudron, JID, 2022, https://pubmed.ncbi.nlm.nih.gov/35485458/;

Jaha, J Antimicrob Chemother, 2023, https://pubmed.ncbi.nlm.nih.gov/37545164/;

Jörimann, JID, 2023, https://pubmed.ncbi.nlm.nih.gov/37498738/

Nevertheless, we fully agree that the bioinformatic pipelines themselves have to be parametrized by the user accordingly to account for the amplification method and NGS technology. This individual parametrization is fully supported by the database.

To clarify this, we made the following changes:

Line 21
We removed “easily” in the abstract.

Line 239f
We added the underlined part in the “SHCND workflow”-section of the methods: “Once the user confirms the selection of NGS records, tool, and relevant parameter settings (appropriate for the NGS record, e.g., NGS platform and amplification method), the data files are transferred to an external high-performance-computing cluster, where they are processed.”

## 2. NGS platform variability:

There is no discussion of how the choice of NGS platform influences the analysis. Pipelines optimised for Illumina, PacBio, or ONT platforms can differ substantially, and this is not addressed in the manuscript.

The sequences in the database are currently mostly obtained by Illumina MiSeq. The pipelines used are appropriately chosen for this. However, we agree that other sequencing methods have their own optimised pipelines. As of now we have mostly Illumina sequencing data, as such we are not employing other pipelines. Nevertheless, the set-up of the database allows a quick implementation of dedicated pipelines if it becomes a necessity. We made the follow changes in the manuscript to avoid confusion:

Line 172f

We added the underlined part in the “Aim”-section of the methods: “Currently the SHCND handles mostly NGS data from Illumina currently. However, the SHCND is designed with a modular and extensible design, allowing for the convenient incorporation of new bioinformatic pipelines and data, particularly when it is available in a containerized from.”

Line 239ff

We added the underlined part in the “SHCND workflow”-section of the methods: “Once the user confirms the selection of NGS records, tool, and relevant parameter settings (appropriate for the NGS record, e.g., NGS platform and amplification method), the data files are transferred to an external high-performance-computing cluster, where they are processed.”

Line 596ff

We added the following sentence in the discussion: “Currently, most sequences in the database were obtained by Illumina MiSeq. The pipelines currently in use are designed for Illumina data. If it becomes a necessity to process sequences from other NGS technologies, the set-up of the database allows a quick implementation of other pipelines.”

## 3. Use of unique molecular identifiers (UMIs):

While some research groups employ UMIs to reduce error and bias, it seems the described pipeline is not designed to accommodate sequences tagged with UMIs. This limitation warrants mention. Without UMIs, the claim that NGS "accurately encapsulates within-host diversity by characterising HIV subpopulations" is likely overstated.

Until now, we did not have the need for pipelines which account for UMIs. However, the database is able to quickly implement new pipelines in a plug-and-play fashion. The claim "accurately encapsulates within-host diversity by characterising HIV subpopulations" comes from the assumption, that with enough sequencing depth, we are able to sequence a large fraction all mutations present in the diverse intra-host viral population. It is correct, that this doesn’t allow us to directly capture, for instance, haplotypes, however, other pipelines are available for this (e.g., VILOCA, Fuhrmann, NAR Genom Bioinform, 2024, https://pmc.ncbi.nlm.nih.gov/articles/PMC11616694/). Finally, in a previous publication, we showed convincingly that UMIs are not necessarily required to study complex virus populations (Seifert, J Mol Biol, 2016, https://pubmed.ncbi.nlm.nih.gov/26711506/)

## 4. Clinical versus research applications:

It is unclear whether the pipeline described is employed for clinical drug resistance testing, and if so, how it differs from its use in research. Clarifying this distinction would enhance the manuscript.

Yes, the MinVar pipeline is used in the diagnostic setting from the Institute of Medical Virology in Zurich. The database currently focuses on the use of NGS data for research purposes. Of course, this does not exclude the use of pipelines which are dedicated for diagnostic purposes and the database could be rapidly adapted for a diagnostic setting.

Line 468

We added the following sentence to the description of MinVar for clarification: “This pipeline is also used in diagnostic settings and was validated accordingly (30).”

## 5. Quality control challenges:

Quality control is only briefly touched upon, with a passing reference to the Hypermut programme. This is particularly relevant for sequences derived from proviral DNA, where maintaining sequence integrity poses significant challenges.

We agree that quality control is an important issue. Basic quality control of the NGS runs is performed upstream of the database by technicians on the Illumina MiSeq machine. In addition, for all our analyses we address quality control in the respective publications listed in Table 3. Finally, the database allows to specify parameters for quality control, individually for each sample and processing pipeline.

Regarding proviral DNA we performed also single provirus sequencing. In these specific cases we used individual parameter choices to check if the sequences are intact (Jörimann, JID, 2023, https://pubmed.ncbi.nlm.nih.gov/37498738/) . In other cases, for example, for viral GWAS studies we assume that viral mutations in defective proviral sequences, with the exception of hypermutations, in a vast majority are also present in the replication competent reservoir. We showed this for example in Neuner-Jehle, PLOS Path, 2024 https://pmc.ncbi.nlm.nih.gov/articles/PMC11335148/, where associations between HIV variants and HLA were similar, if we only included plasma RNA sequences or both plasma and proviral sequences.

In summary, while the manuscript offers valuable insights, it would benefit from a clearer focus on the practical relevance of the described methods, with additional attention to the amplification methods, NGS platforms, and quality control measures essential for both clinical and research contexts.

We thank the reviewer very much for the helpful insights. The comments helped us in making the aims of the manuscript clearer. To summarize, we want to emphasize, that the aim of our database is a platform which provides utility for NGS data processing, originating from different technologies and methodologies and for different research purposes. Currently, it is not the aim of this database to be used for diagnostics. If required, it could be adapted also for this task but would have to be validated accordingly to regulations for diagnostic tests.

# Reviewer #2:

The study presents the innovative solution of SHCND in a sequential easy to follow fashion. I find the methods used detailed in a well streamed flow. The results presented a promising solution to address challenges in the HIV genome data management. Especially in standardizing the steps in handling the data, processing them and retrieving them for research or follow up purposes. The solution presented deserves population to set an example for wider global implementation and further modification to fit the diversity of the features of HIV infection, and might even be applicable for other common infectious diseases world wide.

We are very grateful to the reviewer for the positive feedback.

In this context, I would suggest adding the prospects of widely sharing the achieved goals of the solution and the opportunities of collaboration to widely develop and implement the database solution.

Thank you, we added the following underlined sections in the discussion:

Line 598f

“Moreover, as the core architecture is very general and not specifically tailored to NGS data and bioinformatic workflows, it allows for possible future expansions to include other data types. As such, we believe, that our database solution would be beneficial in many other research settings where complex data is handled.”

Line 603ff

“Currently, our database already includes few Hepatitis C virus (HCV) genomes and the flexible database design would support seamless expansion. Furthermore, a separate instance of the database is currently being set-up for bacterial genomic data and corresponding analysis pipelines, with the goal of conducting real-time molecular epidemiolocal analyses to optimize infection control and facilitating genomic comparisons to better understand resistance spread mechanisms and support targeted interventions.”

One modification to be suggested is the presentation of the references in the introduction, methods and results sections. The citation within the text is distracting, and I would suggest that citation with putting the number of the cited reference in the text. Especially when citing multiple references.

We agree and reformatted the referencing to the journal style.

The study also mentioned that some sample categories were over-represented without giving precise details about which categories are overrepresented and upon the expense of which underrepresented categories.

We added the underlined sections in the relevant paragraph of the results:

Line 496ff

“While the SHCND covers all major demographic groups, transmission modes, and HIV-1 subtypes, several demographic groups among SHCS participants are currently overrepresented, due to the fact that NGS was performed in the framework of specific projects (8,43,53,54,77–80): PWH with any sequence (proviral DNA or plasma RNA) in the SHCS are on average more often white (76.7% NGS vs 64.6% no NGS) leading to an underrepresentation of ethnical minorities (3.4% NGS vs 18.9% no NGS), have HIV-1 subtype B (64.4% NGS vs 47.1% no NGS) leading to an underrepresentation of rare subtypes (16.6% NGS vs 38% no NGS), and were enrolled into the SHCS more recently (median calendar year 2005 NGS vs 1997 no NGS). Additionally, they are more likely to have acquired HIV via homosexual contact (45.2% NGS vs 37.5% no NGS) and less likely through intravenous drug use (10.5% NGS vs 17.1% no NGS) (Table 2).”

The references that presented the preparation, amplification, and sequencing protocols were cited. However, the matching and comparison of these protocols in this study and in the cited references were not detailed.

We revised the following paragraph with the underlined sections to make it more immediately obvious to the reader, what each reference refers to:

Line 177ff

“The SHCND includes data from samples requested for various research projects from centers all over Switzerland, which are shipped for sequencing primarily to the SHCS Laboratory in Zurich and in some cases internationally, for example, to the Sanger Institute in Oxford (8). NGS of near full-length HIV-1 sequences is performed either from plasma RNA or from proviral DNA. The preparation, amplification, and sequencing protocols are described elsewhere in detail for plasma RNA (42,51) and proviral DNA (52,53).”

Finally the language used could be made easier for lay reader, which is something that I always advocate in reviewing any publication, because I believe it will not only benefit reader, but also benefit the published study in giving it wider outreach

We changed the wording at several positions in the manuscript. Particularly the following underlined sections in the introduction:

Line 58f

“…notably to determine: drug resistance mutations (DRM) against anti-retroviral therapy (ART), viral transmission networks between people with HIV (PWH), viral pathogenesis, comorbidities linked to HIV-1, and immune responses, e.g., antibodies against HIV or auto immune diseases…”

Line 64f

“…Sanger sequencing was used to sequence at least the *pol* region (encoding for proteins relevant for viral replication)…”

Line 65f

“…next-generation sequencing (NGS), with technologies such as Illumina and Nanopore,…”

Line 68ff

“NGS has several advantages over Sanger sequencing, especially the ability to detect (resistance) mutations present at low frequencies, i.e., when the mutation is only present in few percent of viral particles (39–41), and to determine within-patient diversity of HIV-1 genotypes as a marker of HIV-1 time since infection, transmission (if viral strains of two PWH are of high similarity), and super-infection (presence of two unique HIV-1 strains from independent transmissions)…”

Line 99ff

“…non-deterministic analyses (processes which have different outcomes despite the same input due to randomness in the algorithms), for example, the subsampling of NGS reads. While modern software mostly allows specifying a so called random “seed” to…”

In addition, as suggested by Reviewer 3, we added two figures (Figure 2 and 3) and one table (Table 1), which explain how the database handles bioinformatic pipeline processing and storage. Finally, the benefits of UUID naming are now explained (Line 194ff).

# Reviewer #3:

Please see comments/suggestions

Introduction

1. The revised version should use PLOS Digital Health's citation style.

We agree and changed it accordingly to the journal style.

2. Paragraph 2 should be broken down into 2-3 paragraphs. The general public may have a difficult time digesting this.

We split the paragraph as suggested.

3. Paragraph 2 shows some obstacles in setting up HIV-related databases but does not provide the specific databases on where these obstacles come from. Are there existing databases (whether HIV-related or not) that you can cite to justify why your database is superior?

We actually observed these challenges in the lack of a database in the day-to-day work within our institution. Most researchers stored and analysed the same sequences individually. The database was meant to overcome these redundancies, not replacing a current database. We emphasized this in Line 186ff: “The steps in the workflow described in Figure 1 were significantly centralized and streamlined with the introduction of the SHCND – prior to its development, similar steps were carried out by each researcher individually with NGS raw reads files stored on a networked file share.”

4. I feel that part of the intro should include the extent by which the development and deployment of the NGS database adheres to FAIR Guidelines: <https://pmc.ncbi.nlm.nih.gov/articles/PMC4792175/>

## We thank the reviewer for this comment. We added the following paragraph in the method section:

Line 142ff

“The SHCND follows the FAIR (Findability, Accessibility, Interoperability, and Reusability) Guiding Principles for scientific data management (51). In brief, it makes data Findable and Accessible, by assigning globally unique and persistent (immutable) identifiers (F1, A1) and by use of standardized HTTPS communication protocols to handle access and authentication (A1.1, A1.2). It uses standard JSON format for rich and extensible metadata capture (F2, F3, A2), which is transformed to an indexed and searchable graph database representation (F4). The system is Interoperable with freely available analysis and processing solutions, specifically targeted at NGS data processing (I1, I3). The database is Reusable with management of data provenance and reproducibility of results, and we argue that reuse of our data and metadata is limited only by medical data privacy (R1, R1.1, R1.2). We also make the case that the infrastructure and design of the system are reusable for other similar efforts and give an outlook to some of the many possible extensions, uses and existing reuses of the system.”

## Methods

5. Most of the arrowheads in Figure 2 are so small that it is difficult to determine their direction. Moreover, just use the full term for BioInf unless you plan to place a legends section.

We adapted the figure accordingly.

6. Please provide a citation for the advantages of using UUIDv4

UUIDs are universally used as identifiers. UUIDv4 is the only version which is truly randomly generated. This is in our case of high importance, to minimize leakage of patient data. We added the following paragraph, including several citations, in the manuscript:

Line 194ff
“The use of UUID version 4 identifiers (55) ensures globally unique, unequivocal, context-free, immutable identification that can be auto-generated in a distributed fashion as opposed to e.g. sequential integer IDs or human readable names, free from any privacy or ordering concerns. UUIDs are in widespread use in technical and non-technical domains for physical and digital artifact identification (e.g., (56–58)). Their special notation makes them particularly easy to recognize and parse as identifiers and we use this extensively to allow users to copy-paste lists of such UUIDs that can be separated by any or no characters at all.”

7. Figure 1 does not show this process: "All generated downstream data files receive their own UUID as well and are linked to the corresponding NGS record and processing tool through additional metadata files." I also feel that Figure 1 needs to be expanded to provide a complete description of the workflow involved in using the database.

We thank the reviewer for noting this. We meant downstream from a NGS record (fastq file) within the database, i.e., data files generated from bioinformatic pipelines. This was of course misleading, as the “downstream” in Figure 1 means downstream of the database, which is no longer part of the database naming conventions. We adapted the sentence to the following:

Line 201f

“All generated data files on the database receive their own UUID as well and are linked to the corresponding NGS record and processing tool through additional metadata files (Figure 2B).”

In addition, we added two other figures and one table to explain the database workflow better.

8. The manuscript will benefit from the addition of additional figures that visualize the contents of each methods sub-section. For now, only the workflow has a visual (which in itself seems to be lacking too).

We added the following additional figures/table**:**

As Figure 2: Illustration of the NGS storage (A) and Bioinformatic result storage (B) implementation in SHCND.

As Figure 3: Illustration of the bioinformatic pipeline processing.

As Table 1: Illustration example of NGS record storage

9. Is the data availability statement found online?

The data availability statement is adapted from the statement of the Swiss HIV Cohort Study (<http://www.shcs.ch/294-open-data-statement-shcs>). As the database is a subunit of the SHCS the same regulations apply.

We added the following sentence in line 490f to indicated this: “The full statement, from which the present statement is derived, can be found online: <http://www.shcs.ch/294-open-data-statement-shcs>.”

Discussion

10. The results do not reflect the statement in paragraph 1 of the discussion section. Can you provide evidence that it's use makes the workflow efficient? While the technical details are presented in the methods section, using the term efficient is farfetched since no information was presented before and after.

We agree that this statement was not factually correct considering the results. While we indeed observed improvements in efficiency, we are unable to quantify this. We changed the sentence (Line 556f) to: “Overall, established in early 2018, the SHCND has streamlined and standardized viral genomic analyses in the Swiss HIV Cohort Study and the Zurich Primary HIV Infection Cohort Study.”
